# Iranian scientists and French showers: collaborative fact-checking of identity-salient online information

**DOI:** 10.3389/fpsyg.2023.1295130

**Published:** 2023-11-09

**Authors:** Eli Gottlieb, Michael Baker, Françoise Détienne

**Affiliations:** ^1^Institut Interdisciplinaire de l'Innovation, Centre National de la Recherche Scientifique, and Télécom Paris, Palaiseau, France; ^2^Graduate School of Education and Human Development, The George Washington University, Washington, DC, United States

**Keywords:** fake news, fact-checking, social media, media literacy, identity, opinion change, collaboration, deliberation

## Abstract

In this study, we investigate what leads people to fact-check online information, how they fact-check such information in practice, how fact-checking affects their judgments about the information’s credibility, and how each of the above processes is affected by the salience of the information to readers’ cultural identities. Eight pairs of adult participants were recruited from diverse cultural backgrounds to participate online in joint fact-checking of suspect Tweets. To examine their collaborative deliberations we developed a novel experimental design and analytical model. Our analyses indicate that the salience of online information to people’s cultural identities influences their decision to fact-check it, that fact-checking deliberations are often non-linear and iterative, that collaborative fact-checking leads people to revise their initial judgments about the credibility of online information, and that when online information is highly salient to people’s cultural identities, they apply different standards of credibility when fact-checking it. In conclusion, we propose that cultural identity is an important factor in the fact-checking of online information, and that joint fact-checking of online information by people from diverse cultural backgrounds may have significant potential as an educational tool to reduce people’s susceptibility to misinformation.

## 1. Introduction

Combating misinformation is one of the greatest societal challenges of our times. In the age of “deepfake,” it is almost impossible for the untrained to distinguish fabricated documents, photos, and videos from genuine ones. Even highly educated individuals, including experts in textual analysis, are poor judges of the reliability of online information (Wineburg and McGrew, 2019). Worse still, research indicates that fake stories travel six times faster and further on social media than do factual ones (Vosoughi et al., 2018). A growing body of evidence suggests that misinformation on social media contributes to political polarization (Guess et al., 2019), affects individuals’ behavior in areas as diverse as voting, vaccination, and recycling (Lewandowsky et al., 2017), and is often believed long after it has been corrected (Wood and Porter, 2019). The most worrying long-term consequence of misinformation, however, may be its erosion of trust in institutions, media sources, science and expertise (van Der Linden et al., 2020). Repeated exposure to misinformation increases the tendency to believe fake news (Pennycook et al., 2018), which is associated, in turn, with a propensity to reject information from expert authorities (Uscinski et al., 2020). This rejection of expert authority deprives people of an important method for distinguishing reliable from unreliable information, thereby deepening the initial problem and undermining efforts by policymakers and educators to address it.

In this study, we set out to address this challenge by investigating what leads people to fact-check information on social media and how these processes are affected by the salience of the information to readers’ personal and cultural identities. Drawing on research in cultural psychology, cognitive science, and human-computer interaction, we use an experimental but ecologically valid situation – collaborative fact-checking of suspect tweets – to investigate how pairs of participants from diverse cultural backgrounds search jointly for information online to evaluate the credibility of claims disseminated on social media.

## 2. Scientific background

Efforts to combat misinformation are only just beginning to come to terms with the scope of the problem (Barzilai and Chinn, 2020). A common approach thus far has been to design or expand programs to develop people’s generic skills of critical thinking. However, dealing with online information often requires quite different skills to those required to read offline texts — for example “critical ignoring” rather than “close reading” (Kozyreva et al., 2023). Accordingly, many current interventions, which focus on generic critical thinking skills rather than skills specific to online texts, such as critical ignoring and lateral reading, may be not only inadequate but perhaps even misdirected (Wineburg and McGrew, 2019; Ziv and Bene, 2022).

One problem is that, while research has provided insight into the judgments people make about the credibility of online information, few studies have examined in detail the processes by which they arrive at such judgments. To date, much research on the role of the Internet in propagating misinformation has focused on the logical structure and vectors of dissemination of conspiracy theories and fake news (e.g., Vosoughi et al., 2018; Ecker et al., 2022) or the characteristics of credulous readers (e.g., Baptista and Gradim, 2022). Few studies have examined how people deliberate in practice about the credibility of information they encounter online (Bago et al., 2020); and those that have done so have tended to define deliberation operationally as silent thinking performed solo (in a figurative “black box” to which the researcher has no access), often in laboratory settings far removed from the contexts within which people normally encounter online information. This has led to calls for more naturalistic studies (Bago et al., 2022, p. 10).

Particularly lacking are studies that examine how people’s identities affect their deliberations about the credibility of online information. Studies of polarization in the consumption and retweeting of online news indicate that people tend to seek out, and to share, news from sources whose political views echo their own (Garimella and Weber, 2017; Jurkowitz et al., 2020). But these studies do not investigate how people assess the credibility of claims they encounter online when the identity of the source is unfamiliar or unclear. Nor do they compare the processes by which people from different cultural groups (e.g., national, ethnic, religious) assess online claims regarding their own cultural group versus claims regarding other cultural groups.

Previous research indicates that people’s cultural identities influence the standards of credibility they use to evaluate claims – a phenomenon known as “epistemic switching” (Gottlieb and Wineburg, 2012). This phenomenon is related to confirmation bias (Tversky and Kahneman, 1974) and cognitive dissonance (Festinger, 1957), in which people process information in ways that maximize its fit with their prior beliefs. However, in epistemic switching, it is not the evaluation of a specific claim that is adapted to cohere with the person’s prior beliefs but rather the evaluative criteria themselves (Gottlieb and Wineburg, 2012, p.11). Thus far, research on epistemic switching has been limited to the evaluation of offline textual information (Gottlieb and Wineburg, 2012). No studies to date have investigated whether or how epistemic switching occurs in the evaluation of online information.

Metzger (2007) argued that assessment of the credibility of online information should not be seen as a single evaluative process but rather as a form of dual processing (Chen and Chaiken, 1999), in which internet users’ accuracy goals vary depending on their motivation for seeking information. To explore this dual processing model, Metzger (2007) proposed viewing credibility assessment as a three-phase process in which exposure to online information is followed by evaluation, which is followed in turn by judgment. According to this model, it is at the exposure phase that motivations are most relevant, as they determine whether, and to what extent, the individual proceeds to the evaluation phase. However, to the best of our knowledge, this model has not been tested empirically. Research is required, therefore, to determine whether the processes by which people assess the credibility of online information follow the phases hypothesized by Metzger (2007) and to what extent the cultural identities of the assessors and the identity-salience of the information motivate particular kinds of evaluation and judgment.

## 3. Research questions

The goals of the present study are to explore:

1. What leads people to fact-check online information;
2. How they fact-check in practice;
3. How such fact-checking affects their judgments about the information’s credibility; and
4. How the above processes are affected by

- the salience of the information to readers’ cultural identities; and
- collaborative fact-checking with people whose identities differ from their own.

## 4. Materials and methods

### 4.1. Methodological approach

To investigate these questions we designed a situation in which pairs of participants collaborate online in selecting specific examples of online information (in this case, Tweets) that they agree to verify together, and which they then verify by searching on Google.

A short list of six (6) tweets was carefully chosen to be on the cusp of credibility (not too obviously true or false) and to relate in some way to the cultural identities of the participants. By cultural identities, we mean, following Tajfel (1974, p. 69):

that part of an individual’s self-concept which derives from his knowledge of his membership of a social group (or groups) together with the emotional significance attached to that membership.

We use the term cultural identity rather than social identity because we focus here on a particular subset of social identities, namely, gender and nationality. For the purposes of the present study, we defined cultural identity operationally as a person’s self-categorization in terms of gender and/or nationality (*cf.* Phinney and Ong, 2007). For example, tweets were chosen on issues relating to England and France for pairs containing one person from each nationality.

Our research design sought to create an experimental situation in which processes of fact-checking would be:

a) Observable: The design prompted participants to engage in deliberation on the credibility of online information as a form of publicly expressed dialog rather than as a private form of individual thinking.
b) Natural: The design employed the “constructive interaction method” (O’Malley et al., 1985), wherein participants working in pairs are led to communicate their thinking as a ‘natural’ part of their deliberation. This can be contrasted with individual protocol analysis (Simon and Ericsson, 1984) wherein verbalization can be experienced by participants as an additional “task.” Our method aims to produce dialogs that provide rich, qualitative information on (inter)cognitive processes.
c) Ecologically valid: The most common situation for evaluating online information might be individual reading. However, sharing and discussing online information with others (for example, in “retweeting”) is also an everyday, and ecologically valid, activity.
d) Related to belief change: To investigate how processes of fact-checking affect belief, we draw on a commonly used method in collaborative learning research (e.g., Simonneaux, 2001; Brocos et al., 2022), whereby participants are asked to state their opinions before and after discussion, with differences then being analyzed in relation to intervening dialog processes.

### 4.2. Materials

To maximize ecological validity, all six of the Tweets that we presented to participants were ones that we had accessed on Twitter while designing the research. Each Tweet appeared as a screenshot from Twitter and included the name and icon of the source feed on which it appeared, and the time and date on which it was posted.

### 4.3. Participants

The experimental interaction situation that we developed was as follows: Eight pairs of adult participants were recruited so that they were approximately matched in age and educational background but differed on one or more of the identity criteria gender and nationality. They were invited to participate via Zoom in an online discussion of the credibility of online information, moderated by one of the authors.

Our sampling scheme was purposive: We recruited participants that we believed would have a theoretical bearing on our results. (*cf.* Patton, 2001). To simplify the definition of participants’ cultural identities, we mentioned explicitly when recruiting them that we wanted our sample to include participants from particular national backgrounds and mother tongues, and that we were approaching them as such (e.g., as a French-speaking woman of French origin). We also mentioned to prospective participants that they might be paired with someone from a similar background to their own or from a different background. Our sample of 16 participants included 7 women and 9 men; aged between 30 and 78; from France and England.

We restricted our sample to French and English participants for two reasons: Relevance and clarity. Our research team comprised two English men and one French woman, and research was conducted while the first author, who is English, was a visiting researcher in France. Our interest in exploring the role of cultural identity in fact-checking made nationality a relevant variable to include in our design. Our personal familiarity with differences between English and French culture, and of common stereotypes people from each country have of each other, enabled us to choose tweets that were likely to elicit different reactions from English and French participants. As the analyses below will show, it also enabled us to interpret participants' interactions, whether in English or French, within a broad cultural and intercultural context.

### 4.4. Procedure

The online interaction comprised four stages.

1. Credibility Assessment: First, each participant completed independently an online questionnaire, in which he or she was asked to assess sequentially the credibility of each of the six tweets on a Likert 7-point scale, from “not credible at all” to “totally credible.” After recording their judgments, participants were required to indicate which, if any, of the tweets they would check, and why.
2. Fact-Checking: Next, participants were brought together on Zoom to select two tweets (from the six presented in the first section) that they wished to check together. After selecting the tweets, each participant took turns being the “searcher,” i.e., sharing their screen while entering search terms into Google to check the tweet’s credibility and discussing the results with their partner. When the participants felt they had reached a conclusion about a given tweet’s credibility, they ended their search and each of them entered their conclusions into the online questionnaire.
3. Credibility Reassessment: Then, each participant completed independently, a second time, the online questionnaire, to record their post-deliberation assessments of the credibility of each of the six tweets (i.e., two that they had checked together, and four that they had not checked).
4. Debrief: Finally, the participants were invited by the moderator to comment on their experience and share any general thoughts that occurred to them during the course of the session.

**Figure 1:**
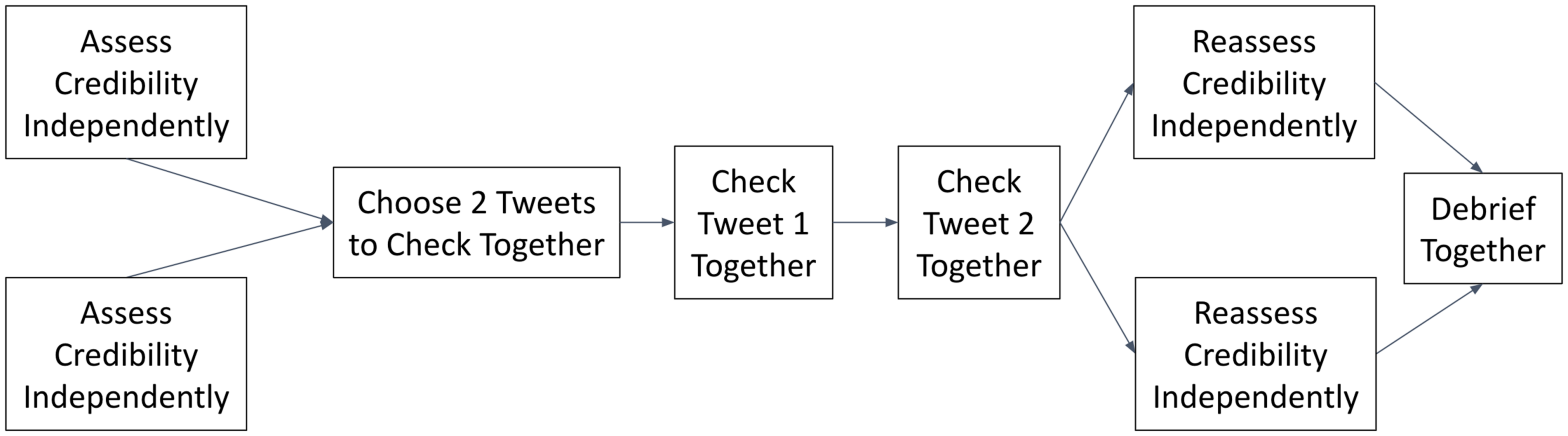
The experimental procedure.

This procedure is summarized in Figure 1.

To investigate the effects of participants’ cultural identities on their joint fact-checking deliberations, we used a classic 2 × 2 design to compare the deliberations of participant pairs that were matched for cultural identity (e.g., English-English) with pairs that were mismatched (e.g., English-French); and deliberations in which Tweet content that was of high identity-salience to the participants (e.g., an English-French pair fact-checking a Tweet about how often French people shower) with those in which Tweet content was of low identity-salience (e.g., an English-French pair fact-checking a Tweet about the percentage of science and engineering graduates in Iran that are women.)

**Figure 2:**
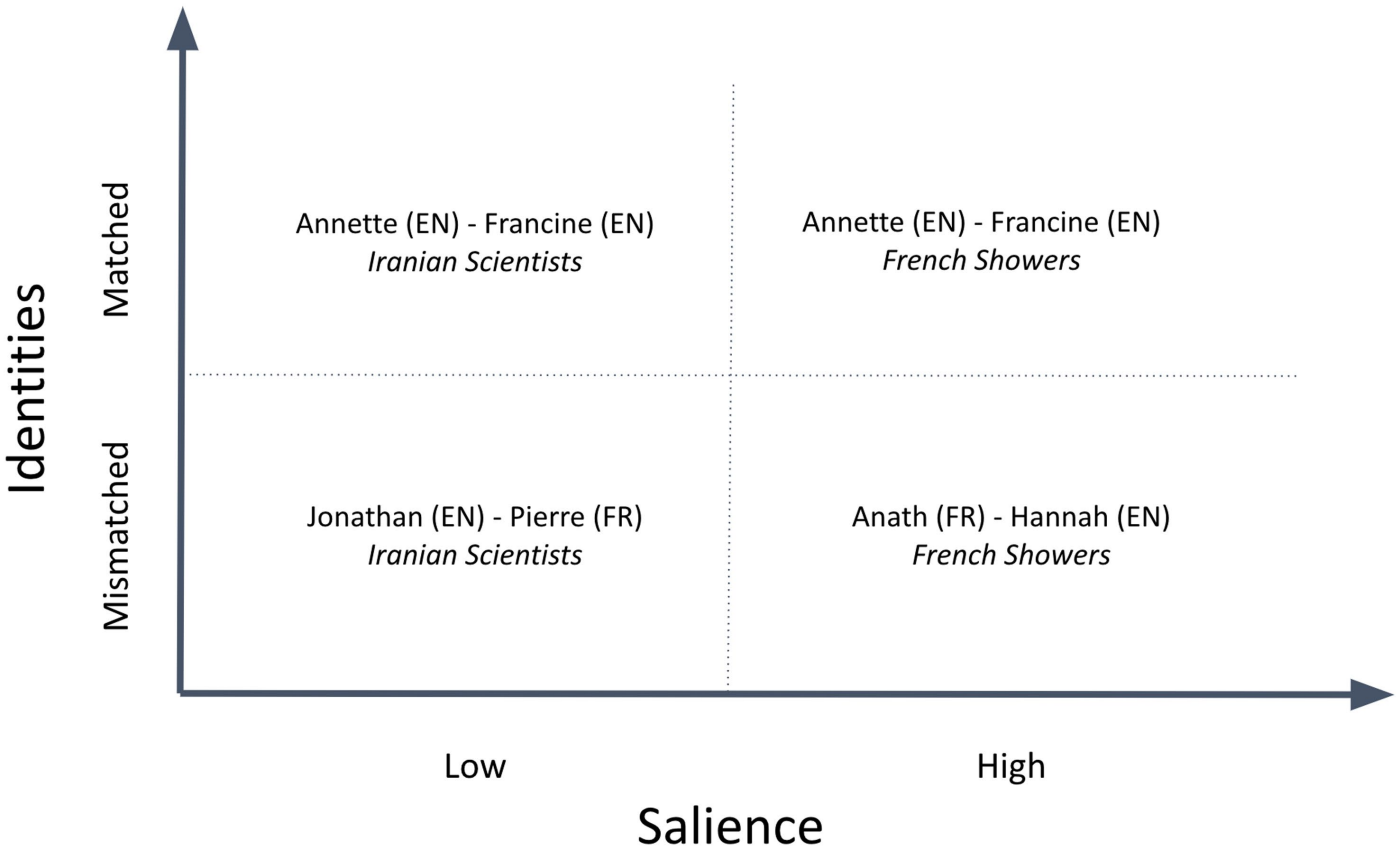
2 × 2 experimental design to investigate identity and salience effects, including sample deliberations selected on this basis for detailed analysis.

This 2 × 2 design, summarized in Figure 2, includes examples of fact-checking deliberations in each quadrant. We analyze each of these examples in detail in the results section.

### 4.5. Coding scheme

The Zoom sessions were recorded and transcribed verbatim. Coding categories were derived inductively, using an iterative procedure in which two of the authors each coded a transcript independently and devised a set of categories sufficient to account for fact-checking processes contained therein. After completing their initial coding, the coders met to compare categories and construct a coding scheme on which they could both agree. Each then employed this new coding scheme to code independently a second transcript. They then met again to compare their results and further refine the coding scheme. After a third iteration the coding scheme was tested formally for inter-coder reliability on four transcripts (i.e., 25% of the total corpus). Inter-coder agreement was high, Cohen’s κ = 0.91. Disagreements were resolved by discussion and the remaining transcripts coded by a single coder.

The coding scheme, with decision rules and examples from the corpus, is presented in Table 1.

**Table 1:** Coding scheme.

Category	Rule	
Judge	Judging tweet credibility, with or without explicit justification	“The Iranian one sounds fake.”
Decide	Statement or reasoning about whether to check the tweet	“Well, I think the Iranian one might be easy to check.”
Analyze	Analyzing the form and content of the tweet	“Yeah, so they said only 43% of Frenchies are not showering on a daily basis”
Plan	Discussing how to search, what to search for, what to do next	“I’ll try to go straight to the point, otherwise we will look a little more.”
Formulate	Discussing which terms to enter into the Google search, etc.	“Open a new Google page on the side and I tell you what to copy paste in there. Yeah.”
Search	Action, directions, instructions, discussion regarding the search	“This top one?”
Interpret	Interpreting the results of the search, including comparing sources, claims	“Er, that’s about how often do they use a washing machine. That’s something different.”
Evaluate	Evaluating evidence, sources	“Let us see if they say how many people they have sampled, for instance.”
General	General reflections on the activity, online credibility, fact checking	“… things that are politically touchy, you assume some good media has already fact-checked it …”
Other	Not clearly in any of the above categories, anything off-task	“Hmm,” “Wow,” “Yeah,” Etc.
N/A	Ineligible for coding	(Experimenter) “Would you say you have reached a conclusion or is there more that you’d like to check?”

## 5. Results

### 5.1. The decision to fact-check

What leads people to check online information? Our study provided us with two sources of relevant evidence. First, as noted, participants were asked explicitly in the questionnaire to indicate, independently of each other, which, if any, of the tweets they would check, and why. Second, when deliberating about which two tweets they wanted to check together, participants discussed explicitly reasons for checking, or not checking, particular tweets.

#### 5.1.1. Reasons given in the questionnaire for (not) fact-checking

**Figure 3:**
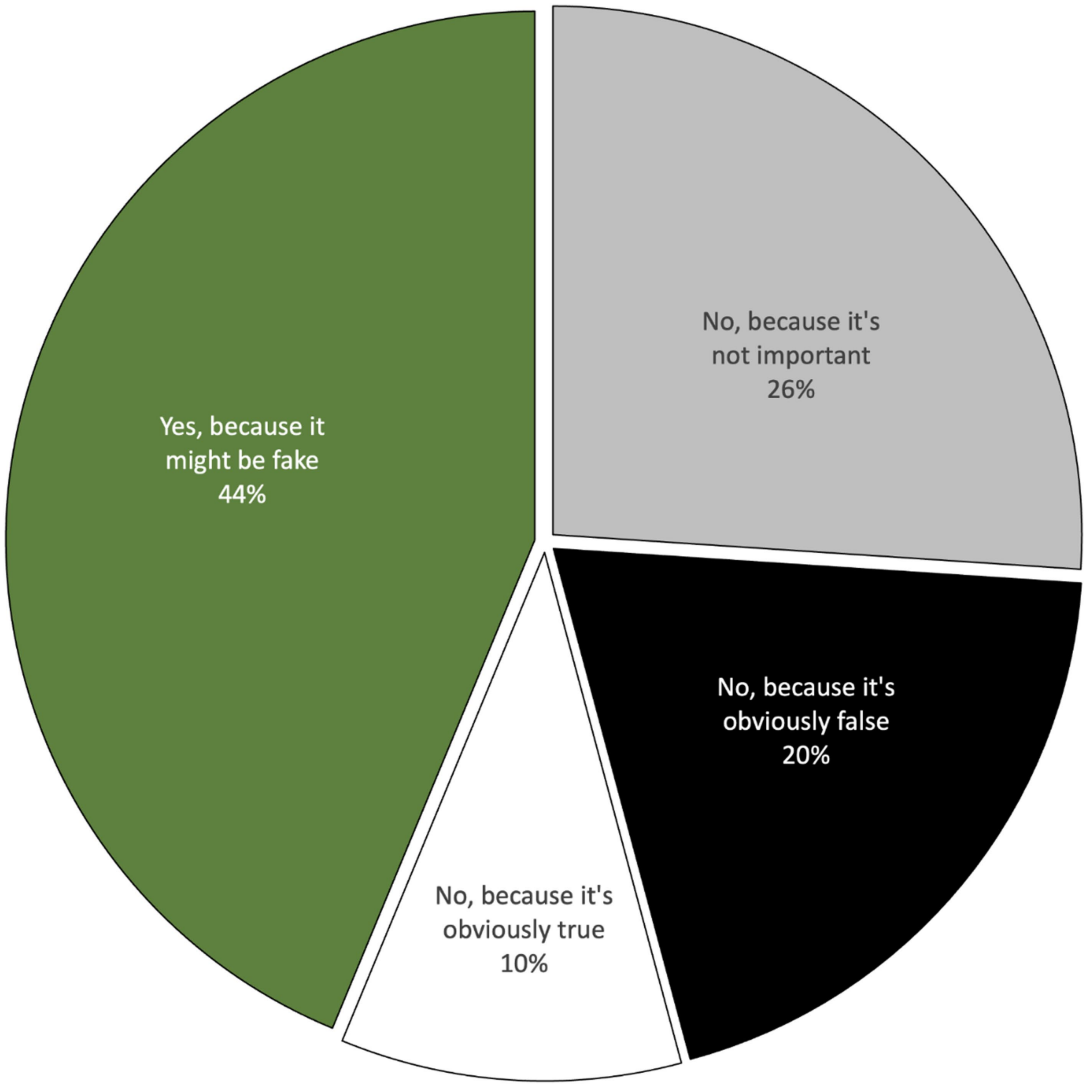
Reasons given for (not) fact-checking (Questionnaire).

As can be seen in Figure 3, participants reported that they would indeed want to check several of the tweets presented in the questionnaire. However, on average, they reported being slightly more likely not to check tweets than to check them (56%). The reason participants gave individually for not checking was more often that the tweet wasn’t important enough to merit further investigation (30%) than that it was obviously true (10%) or obviously false (20%).

**Figure 4:**
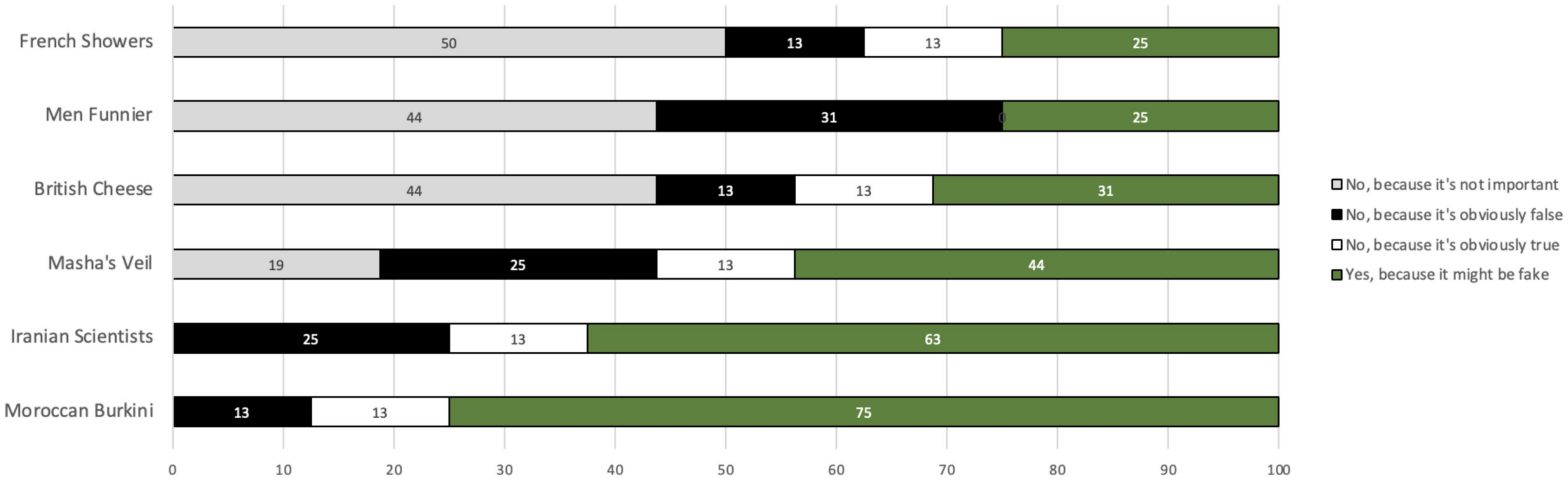
Reasons given, by Tweet, for (not) fact-checking (Questionnaire).

However, as can be seen in Figure 4, some of the tweets were considered more suspect than others. In particular, a majority of participants reported that they’d check the tweets reporting that most science and engineering graduates in Iran are women, and that burkinis are banned in Morocco, because they suspected these might be fake. In contrast, the tweets claiming that only 43% of French people shower daily, that Britain produces more varieties of cheese than France, and that men are funnier than women, were considered by around half of the participants (50, 44, and 44%, respectively) to be insufficiently important to merit fact-checking. Especially interesting is the finding that only a small minority of participants said the reason they wouldn't check a given tweet was because it was obviously true (10%) or because it was obviously false (20%). In summary, it appears that the decision about whether or not to fact-check a given tweet hangs firstly on whether the reader considers the claim worthy of investigation and secondly on the extent to which the tweet seems true or false.

#### 5.1.2. Reasons given during joint deliberation for (not) fact-checking

During their joint deliberation about which two tweets to check together, participants often cited additional motivations for checking or not checking various tweets. Generally, participants cited specific doubts based on prior knowledge or experience. For example, Jonathan, an English professor of political science tells his interlocutor, Pierre, a French professor of management, that he wants to check the tweet about Iranian scientists because it seems to him both plausible and suspect.

I would go for the Iranian women one, the reason being that Iran isn't Saudi Arabia, so women do actually have much more of a normal role than they do in the Arab states and the Gulf. Much more of a public role. [IS-JP-6 (Decide)]

Here, and below, utterance numbers appear in square parentheses following each excerpt, alongside initials indicating the Tweet and the participants, and followed in round parentheses by the relevant coding category. For example, in the excerpt above, Utterance 6 in Jonathan and Pierre’s deliberation about Iranian Scientists appears as [IS-JP-6 (Decide)].

Pierre concurs, citing his own combination of doubt and readiness to be surprised:

So, I doubt it's true, but I could still believe it might be okay. [IS-JP-7 (Judge)]

However, in some cases, participants decided to check tweets about which they were already firmly convinced. For example, in their questionnaires, English participants, Annette and Francine, each rated the tweet about how often French people shower as totally credible. Nonetheless, they still wanted to check it. Before stating why, both participants launched into a series of derogatory remarks about French cleanliness. Annette starts:

Stinky French. It's because of that hole in the floor for toileting, all those years ago, I'm sure. [FS-AF-1 (General)]

Francine responds:

Of course they don't wash. We know they don't wash. [FS-AF-3 (General)]

This sets the (sometimes mildly humorous) tone for the rest of the dialog, which is sprinkled liberally with further comments about “the stinky French.” It is as if Annette and Francine chose to check this particular tweet in order to confirm their prior belief that the French are less than meticulous about personal hygiene.

In other pairs, participants chose to fact-check tweets that related to perceived cultural differences between them. For example, Hannah, an English woman, tells Anath, a French woman, that she’d like to check the tweet about how often French people shower:

Because I feel that's possibly where the English and the French have their most, erm, not our individual interests, but certainly culturally. [FS-AH-11 (Decide)]

Anath agrees, with a hint of self-deprecation (indirectly self-designating as a “Frenchie”)

Yeah, so they said only 43% of Frenchies are not showering on a daily basis. [FS-AH-12 (Analyze)]

Unlike Annette and Francine, Anath and Hannah are unsure initially about the tweet’s credibility. In their pre-deliberation questionnaire responses, Anath gives it a score of 3 out of 7, and Hannah a score of 4 out 7. However, Anath adopts an ironic, defensive posture during the deliberation. For example, immediately after her comment above, she adds:

but who knows what people have corrupted Google to put against French people. [FS-AH-14 (General)]

In summary, our qualitative data from participants’ joint deliberations about which tweets to check suggest that at least one reason that people decide that a given piece of online information is worthy of further investigation is that it pertains to their own cultural identity or their prior beliefs about other cultures.

### 5.2. The checking process

How, in practice, did participants fact-check the tweets they selected?

Our first finding was that, in contrast to Metzger’s (2007) linear theoretical model of credibility assessment of online information (see especially Figure 1 in Metzger, 2007, p. 2088), participants did not, in general, reach a judgment about the credibility of what they had read online after reviewing evidence. On the contrary, their review of evidence and deliberations on its quality and relevance was usually sandwiched between two or more credibility judgments. Typically, their joint deliberation began with an initial assessment of the Tweet’s credibility, to which they then referred when deciding whether or not to check it. This initial judgment was then updated iteratively over the course of the deliberation until a conclusion was reached.

Similarly, the other elements of fact-checking that we identified in our analysis of participants’ deliberations did not generally follow a predictable, unidirectional path. Instead, while all the pairs we studied employed each of the elements, they did not all employ them to the same extent or in the same order. Accordingly, our inductively generated model includes the possibility of multiple pathways and iterations between initial and final credibility judgments.

In other words, the data collected from the joint deliberations of our participants embodied the distinction that Dewey (1902) made between logical and psychological principles of organization (*cf.* Henle, 1962). Theoretically, it may be logical, after deciding to check a Tweet, first, to analyze its form and content to pinpoint precisely what to check; then, to plan how to check it; then, to formulate search terms to input into Google to access relevant evidence; then to interpret the results of the search; then, to evaluate their quality and relevance; and, finally, on the basis of the accumulated evidence, to judge the Tweet’s credibility. However, in practice (i.e., from a psychological perspective rather than a logical one), people’s deliberations are not generally linear. Their start and end points vary, and they include multiple iterations and revisions.

#### 5.2.1. Analytical model

**Figure 5:**
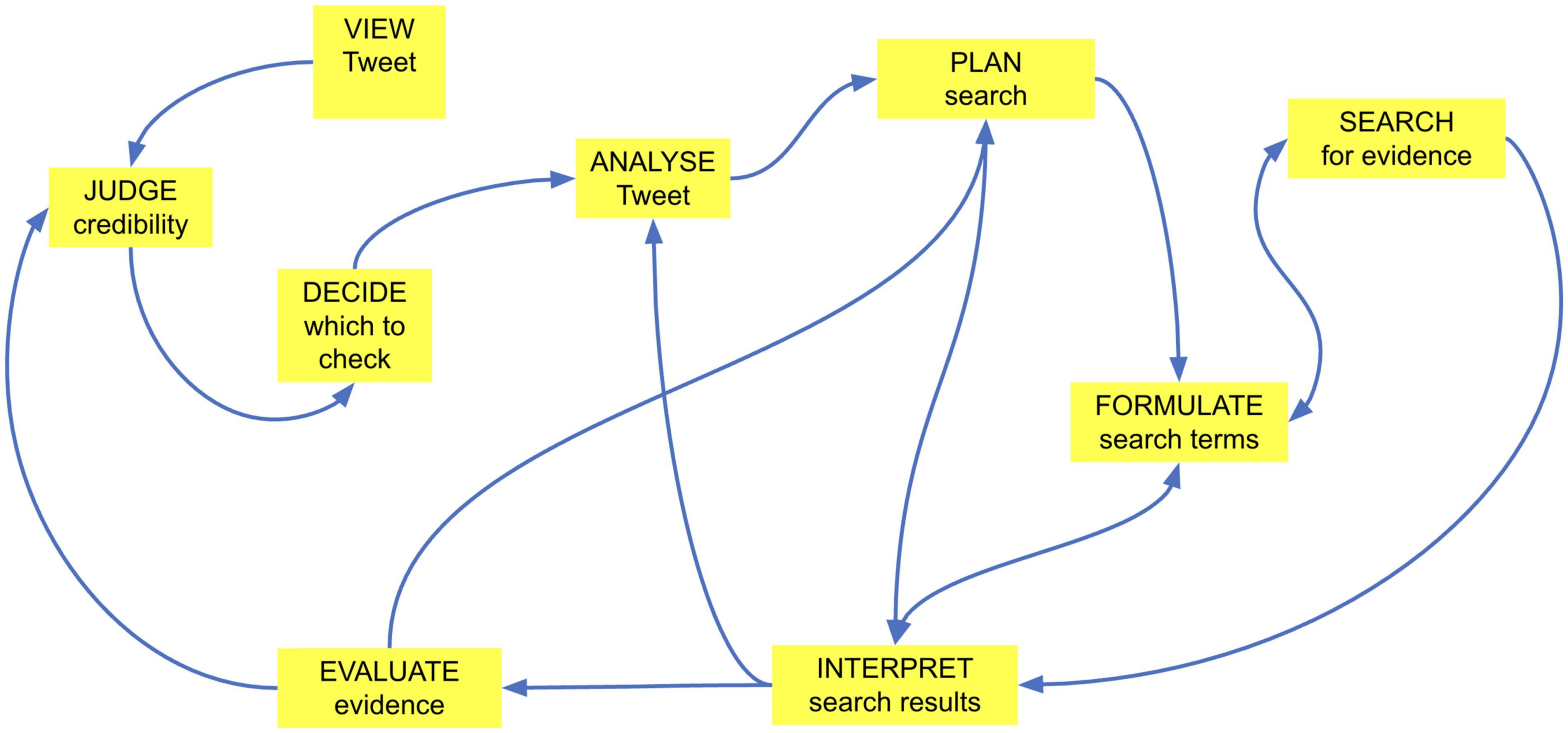
Dynamic analytic model of collaborative fact checking.

We represent these multi-directional and iterative features of fact-checking deliberations in the analytical model summarized in Figure 5. As the arrows indicate, it is possible, and sometimes the case, for the direction of deliberation to follow what might be considered the most logical sequence: View, Judge, Decide, Analyze, Plan, Formulate, Search, Interpret, Evaluate, Judge. However, more often, our participants’ deliberations included loops and jumps. For example, one common loop was: Plan, Formulate, Search, Interpret, Formulate, Search, Interpret. In such cases, after interpreting the results of their initial search, participants reformulated their search terms to seek out other, additional or more relevant, evidence. Similarly, participants sometimes jumped from Plan to Interpret, as they essentially planned, formulated and searched “with their fingers,” typing while talking with their interlocutor about the search terms they were entering into Google, pressing search, silently reading the results – all in a matter of seconds – before interpreting the results of the search.

#### 5.2.2. The shape of deliberations

To explore how this ‘canonical’ analytic model played out in practice in relation to the content of the Tweet being checked and the cultural identities of participants, we devised a method to represent fact-checking deliberations graphically. This involved plotting the categories of deliberation along the y-axis and the series of utterances comprising the deliberation along the *x*-axis.

To illustrate how such graphic representations can be used to compare and contrast collaborative fact-checking deliberations, we present below a detailed qualitative analysis of two such deliberations – the first short and relatively linear; the second longer and loopier. Both deliberations are from mismatched, English-French pairs; but the first is about a low-salience Tweet (Iranian Scientists) and the second about a high-salience Tweet (French Showers).

##### 5.2.2.1. Jonathan and Pierre on Iranian scientists

**Figure 6:**
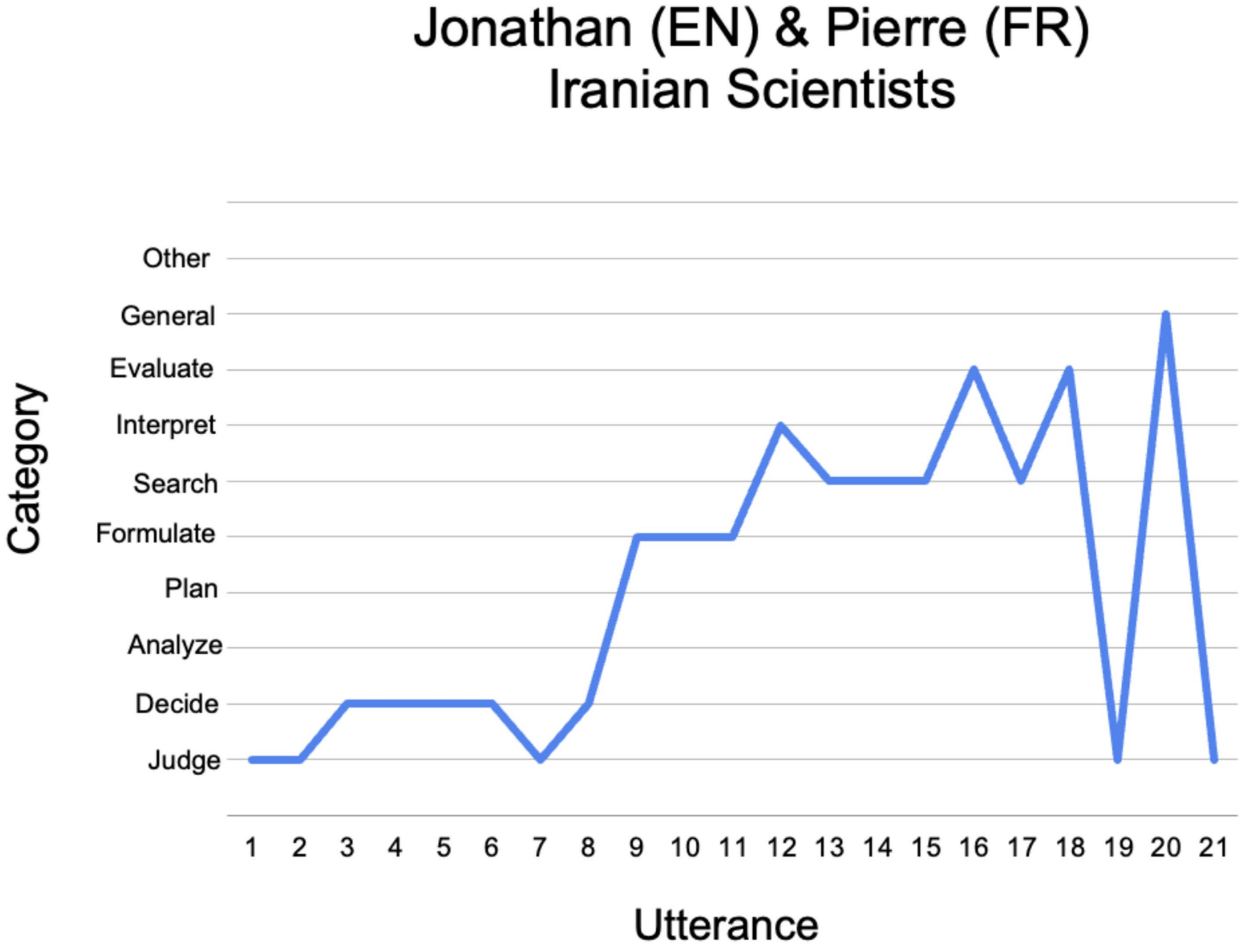
Jonathan (EN) and Pierre’s (FR) fact-checking deliberation about Iranian scientists.

As we have seen already, Jonathan and Pierre’s deliberation (Figure 6) begins with a preliminary judgment that the Tweet is suspect but might be true, and a joint decision to check it. Next, Jonathan, who is the “searcher” in this deliberation, formulates search terms and checks in with Pierre:

Okay. So I'm gonna put in Iran engineering graduates, men and women. Yes? How's that sound?” [IS-JP-9 (Formulate)]

The search results appear to give a clear and positive answer, at the top of the first page of results provided by Google, which Jonathan reads out loud:

Forbes says 70% of Iran's science and engineering students are women. [IS-JP-12 (Interpret)]

However, Jonathan does not simply accept the information provided as coming, as it appears, from Forbes. He opens a new tab and checks that the general appearance of the Forbes website corresponds to the page they have just seen:

Let’s see if it really is Forbes … It's definitely from Forbes. [IS-JP-13-16 (Search, Evaluate)]

Next, Jonathan points out that the information from Forbes is corroborated further down the first page of results of his initial Google search by other sites that he considers to be fairly reliable, such as The Hill, Quora and Reddit. Finally, Jonathan evaluates this evidence as being sufficient:

So I would say, yeah, that would be enough for me. [IS-JP-19 (Judge)]

Pierre concurs:

After I saw it on Forbes, I don't think that I would have looked much forward. I would have probably stopped here and considered that it's true, even if I am very surprised. [IS-JP-20 (General)]

Figure 6 represents schematically these features of Jonathan and Pierre’s deliberation. The deliberation is short, comprising only 21 utterances. Jonathan’s initial formulation of search terms yields a “direct hit,” whereby the first page of Google results linked to an article in Forbes confirming the Tweet’s claim. Jonathan trusts Forbes as a reliable source of information. But to make sure he was not being duped by a site masquerading as Forbes, Jonathan engages in “lateral reading” (Kozyreva et al., 2023), opening a new tab and checking that the format of the Forbes website corresponds to the format of the site they have just viewed.

##### 5.2.2.2. Anath and Hannah on French showers

**Figure 7:**
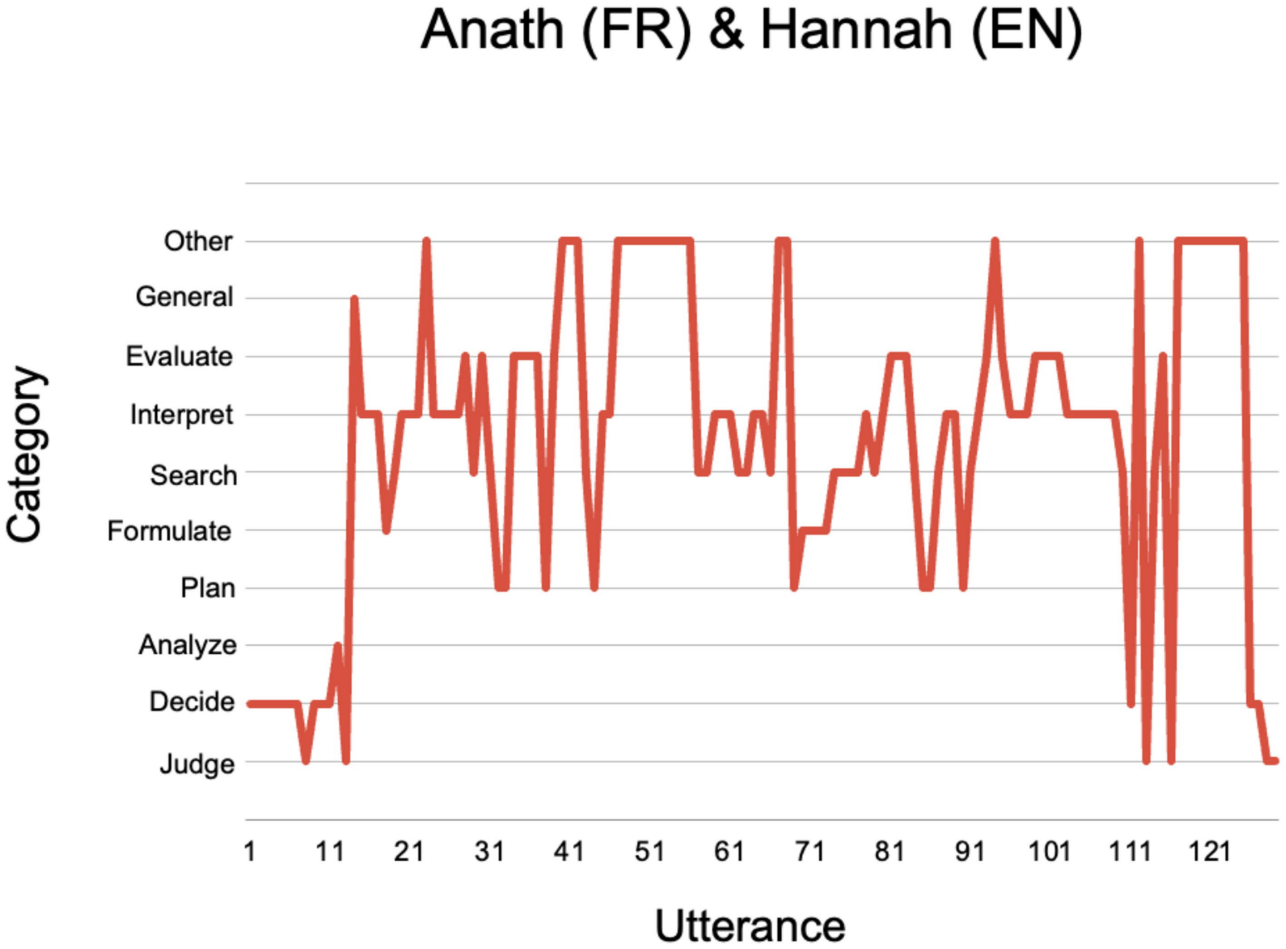
Anath (FR) and Hannah’s (EN) fact-checking deliberation about French Showers.

The shape of Jonathan and Pierre’s deliberation about Iranian Scientists (Figure 6) differs markedly from that of Anath and Hannah about French Showers (Figure 7).

First, Anath and Hannah’s deliberation is much longer than Jonathan and Pierre’s, comprising 147 utterances (vs. Jonathan and Pierre’s 21). Second, it contains more iterations, as the pair plan and re-plan their search, formulate new search terms, interpret the results and seek further evidence. Throughout their fact-checking deliberation, Hannah often turns to Anath as a translator and expert on French culture. Their deliberation also contains numerous diversions and ‘zoom-outs’ as the pair steps back from the specific search to share general thoughts about cultural difference and the relevance of different kinds of evidence.

As we saw earlier, Hannah and Anath’s decision to investigate this Tweet is motivated partly by their interest, as a “mismatched” pair, in exploring together a topic that relates to possible differences between English and French culture.

Hannah takes the role of searcher. She begins by formulating search terms: “how often do French people shower,” inputting them into Google, and pressing the search button.

Immediately after the results appear on the shared screen, Anath begins to interpret them:

That's probably the source of that Tweet. [FS-AH-27 (Interpret)]

Then, right away, she seeks to dig a little deeper:

But is there a proper study for that? [FS-AH-28 (Evaluate)]

Hannah then clicks on a link at the bottom of the first page of results from her initial Google search to an article on the Thrillist website titled, “French people do not like to shower, survey shows.” The article mentions a study conducted by poll company, BVA, and published in French newspaper, Presse-Océan, showing that “only 57% of the French shower daily.”

Hannah asks Anath if she is familiar with the poll company and newspaper. Anath says “no,” but comments that Presse-Océan sounds like a local newspaper from the west of France. Hannah then clicks on the link to the newspaper, which (confirming Anath’s conjecture) connects to a website named Ouest-France, and an article in French titled, “Les Français restent en moyenne 9 min sous la douche” (which translates as: On average, the French stay under the shower for 9 min).

Anath points out that this does not give the answer they are looking for and instructs Hannah to open a new Google page. She then asks Hannah to copy-paste into Google from the Ouest-France article (“BVA-Doméo-presse régionale, sur les français restent … sous la douche” [English translation: “on the French remaining … under the shower”]). This links to the BVA website, the language of which is French, and which Anath helps Hannah to navigate, acting as her interpreter.

Anath: And that's basically, they are saying, that's the study about French people and what are our habits. And this second one.

Hannah: This top one?

Anath: No, the second … Let’s see. And scroll down. Ok, ok so let's see. Scroll down. Yeah, So stop it here, just go a bit further up. They say here as well that only 57% are taking a shower every day. [FS-AH-73-80 (Formulate, Search)]

Having successfully located the source of the 43% statistic cited in the original Tweet, Anath now questions the study’s trustworthiness:

So the question. I'm not sure how trustable this study is. I don't know who they are, if it's a big group or not. I have no idea who this BVA is. If you scroll down to the end of the article, let's see if they say how many people they have sampled, for instance. [FS-AH-86-90 (Evaluate, Search, Evaluate)]

Anath then directs Hannah to click on a link titled “sondage” (English translation: opinion poll). This leads to a PowerPoint presentation containing details of how the study was carried out, including the number and age of participants and their representativity of the French population. Scrolling down to look at the error margins, Hannah notes that it’s 95% reliable, which leads her and Anath to concur that the study appears to support the Tweet’s claim.

Anath: Trustworthy. Given the sampling variety.

Hannah: Yeah, I'd say, if nothing else, it was a serious study. [FS-AH-98-99 (Evaluate)]

Next, Hannah and Anath exchange general remarks about their judgments of the Tweet’s credibility in relation to cultural differences between them. Anath admits to a lingering suspicion about the Tweet’s credibility whereas Hannah acknowledges that, were she fact-checking alone, it would have been enough for her to see that the Tweet was based on a survey, and she would not have dug any deeper into questions of sample size or error margins.

Hannah: I think in truth if I'd seen that, whether I believed in the survey or whatever itself, I would probably stop and at least think the tweet was based on something.

Anath: I was surprised. I would investigate more. And then when I see the study, unless I go and checkup, who is BVA, I would say maybe they've got some interest in saying that. I would say, like, commercial interests … Otherwise, I would say it’s probably a subset of reality; I would tend to believe it.

Hannah: I think the truth is that part of the cultural thing is, my interest in French people showering, to be honest, it's probably slightly less than Anath’s. Because she feels something about her belief in it, whereas for me, it's just a figure. [FS-AH-125-131 (Judge, Search, Evaluate, Judge, General)]

Further evidence of Anath’s greater stake in the Tweet’s credibility, and her corresponding reluctance to take the results of their fact-checking at face value, is her use of irony and self-deprecating humor. During the debrief section of the dialog, Hannah drops off the videoconference due to an internet problem. Echoing her initial defensive posture, Anath jokes: “I hope it’s not because of the French smell!”

### 5.3. Belief change

We have seen that fact-checking deliberations tend to comprise a determinate set of processes (deciding, analyzing, etc) but that the order in which they appear, the extent to which they are employed, and the number of iterations through which go can vary considerably. But what are the effects of such deliberations on people’s judgments of the credibility of the online information with which they were originally presented?

**Figure 8:**
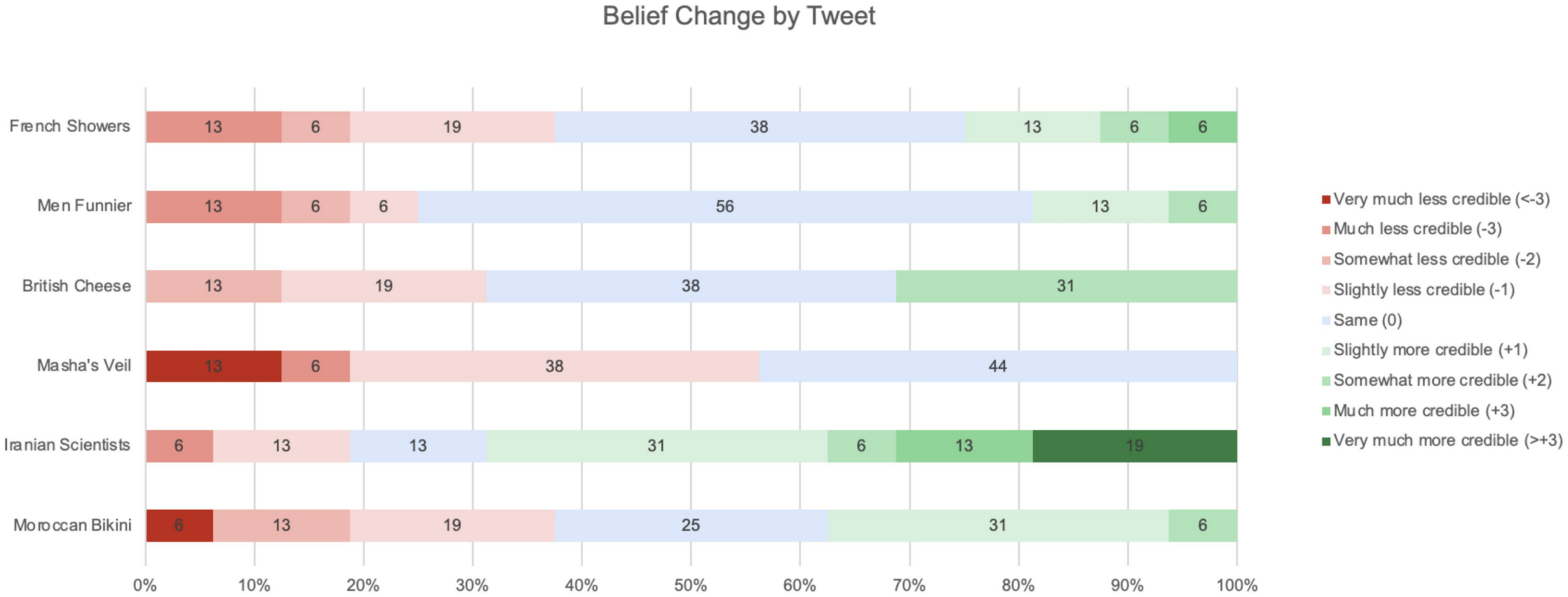
Belief change by Tweet.

To examine this question, we compared participants’ assessments of Tweet credibility before and after deliberation. We calculated the degree of belief change by subtracting participants’ post-deliberation credibility ratings from their pre-deliberation ratings. For example, if a participant gave the Iranian Scientists Tweet a credibility rating of 2 prior to deliberation and a rating of 5 after deliberation, the degree of belief change was calculated as 5–2 = +3. Figure 8 presents the degree and direction of belief change for each tweet.

Each participant rated each tweet twice – once before, and once after, deliberation. Therefore, Figure 8 is derived from a total of 96 credibility ratings (2 per tweet, for 6 tweets, from each of 16 participants).

**Figure 9:**
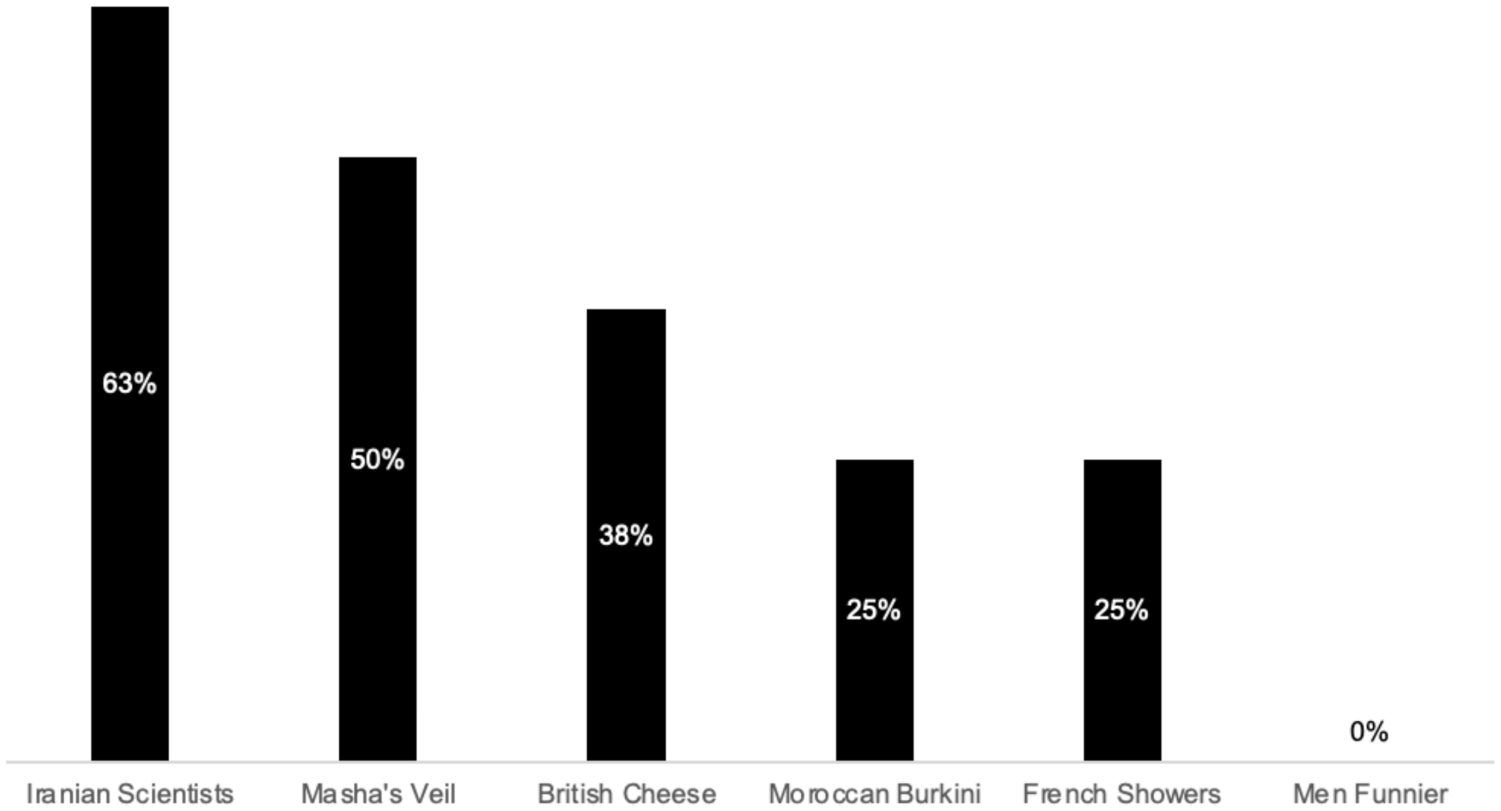
Percent of participants that fact-checked each Tweet.

One striking finding is that there is considerable belief change, in both directions, for all but one of the Tweets. The exception was Masha’s Veil. This was a Tweet purporting to be from French politician Marine Le Pen’s Twitter feed, criticizing a state-run French TV channel for broadcasting a children’s cartoon in which the heroine wears a head covering. The Tweet was considered by most participants to be considerably less credible when it was assessed a second time, even though, as can be seen in Figure 9, only 50% of the participants had fact-checked it in the interim.

This suggests that the act of fact-checking may have a general effect on credibility judgments – including about online information that has not itself been checked. In the case of Masha’s Veil, the effect was negative, i.e., participants became more skeptical about this Tweet’s credibility, even when it was not one of the Tweets they had checked. With all the other Tweets, the effect was both positive and negative, with some participants becoming more skeptical, and others becoming less so, after deliberation.

Further evidence of this general effect of fact-checking on credibility judgments – even of Tweets that have not themselves been checked – is provided by the Men Funnier Tweet. This Tweet, from the British Broadcasting Corporation’s Twitter feed, cited a study claiming to show that men are funnier than women. As can be seen in Figure 8, nearly half the participants changed their assessment of its credibility after fact-checking other tweets, despite the fact that none of them had fact-checked the Men Funnier Tweet itself in the interim (*cf.* Figure 9).

We expected that belief change would be less pronounced for Tweets about which participants were initially suspicious. In practice, however, the opposite was true. For example, 75% of participants reported in the questionnaire that they would like to check the Moroccon Burkini Tweet (a Tweet suggesting that Burkinis are prohibited in swimming pools in Morocco) because it might be fake. However, in practice, this was the Tweet with the greatest degree of belief change, with 37% of participants considering it more credible, and 38% of participants considering it less credible, after deliberation.

Similarly, belief change was just as evident, in both directions, for Tweets initially considered insufficiently important to check (such as French Showers) as for Tweets considered suspect and important enough to warrant checking (such as Moroccan Burkini).

As can be seen in Figure 9, the Tweet fact-checked in practice by most participants was Iranian Scientists. It was also a Tweet that most participants picked out in the questionnaire as one they wanted to check because it might be fake. As we saw above in the analysis of Jonathan and Pierre’s fact-checking of this Tweet, evidence supporting its credibility was readily available, with an initial Google search yielding multiple reliable sources that appeared to back it up.

This combination of initial suspicion and subsequent revelation of supporting evidence is the likely explanation for participants’ anomalously high degree of positive belief change in relation to the Iranian Scientists Tweet. Indeed, it is possible that participants’ experience in relation to this Tweet, namely, of initial suspicions proving unfounded, accounts for at least some of the positive belief change in relation to other Tweets. “If I was overly suspicious about this Tweet,” participants may have thought, “perhaps I’m also being overly suspicious about these other Tweets, too.”

To summarize the findings of quantitative analyses of all participants’ credibility judgments before and after joint fact-checking (presented in Figures 3, 4, 8, 9), fact-checking appears to promote belief change, as a function of the deliberation process. Moreover, the effects of fact-checking appear to extend beyond the particular Tweet checked, affecting credibility judgments about other Tweets, which were not themselves checked.

### 5.4. The role of identity

How were the processes of deliberation and belief change described above affected by (a) the salience of the information to readers’ cultural identities, and (b) collaborative fact-checking with people whose identities differed from their own?

As noted earlier, to investigate these questions, we employed a 2 × 2 design to compare the deliberations of participant pairs that were matched for cultural identity (English-English) with pairs that were mismatched (English-French); and deliberations in which Tweet content was of high identity-salience to the participants (French Showers) with those in which Tweet content was of low identity-salience to participants (Iranian Scientists).

**Figure 10:**
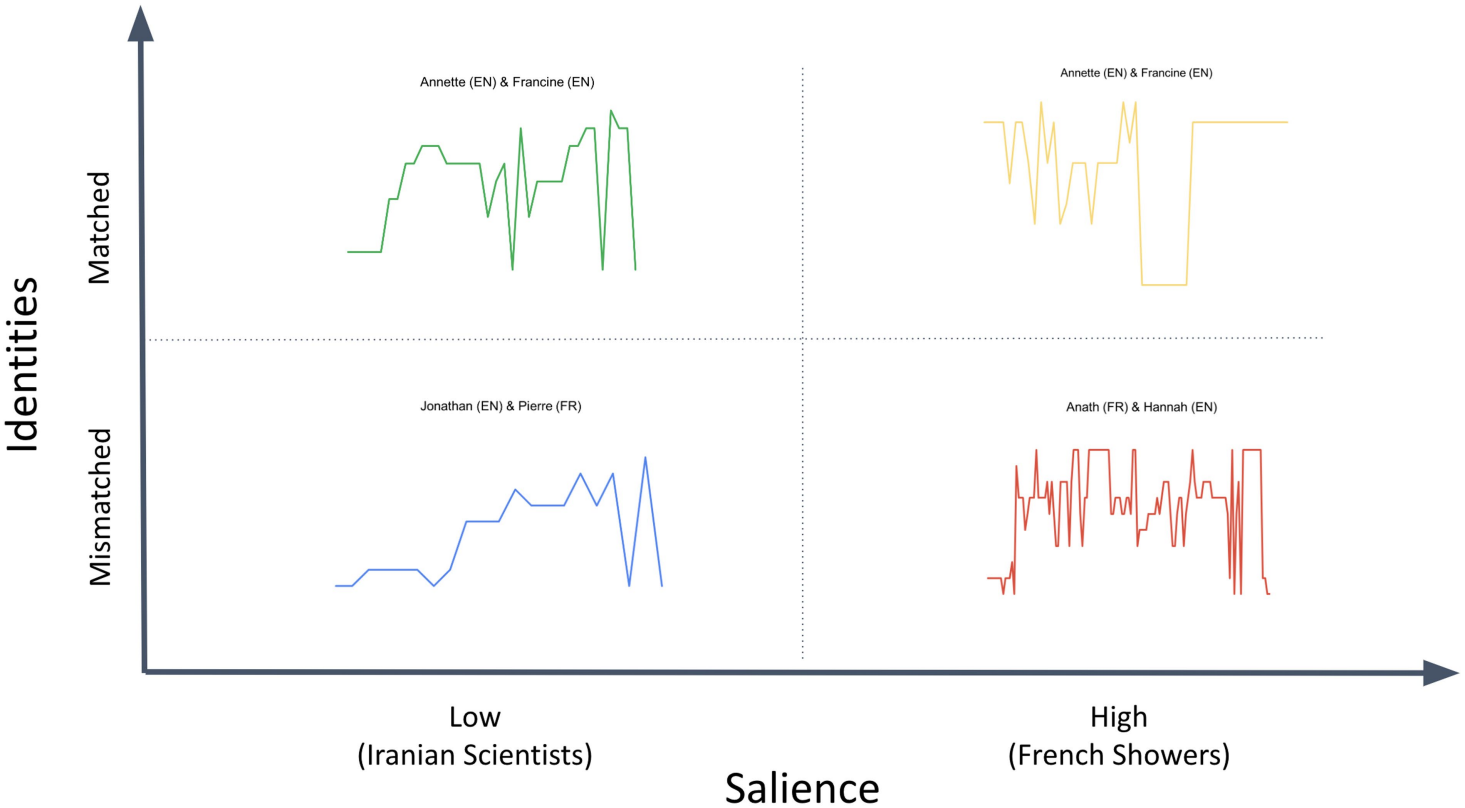
Four deliberations compared.

Figure 10 compares the shapes of four deliberations, using this 2 × 2 design. This graphic representation highlights three intriguing points of contrast.

First, when the Tweets are of low salience to the participants (i.e., the green and blue deliberations on the left side of the figure), there is little difference between the deliberations of the matched pair and the mismatched pair. Both deliberations are fairly linear, with few loops or jumps.

Second, when the Tweets are of high salience to the participants (i.e., the yellow and red deliberations on the right side of Figure 10), the deliberations are longer and more iterative, going more rounds before reaching their conclusion.

Third, these effects of high salience are more pronounced for mismatched pairs than for matched pairs. Specifically, as mentioned in our qualitative analysis earlier, the matched pair’s high-salience deliberation (Annette and Francine on French Showers) focused primarily on confirming their prior beliefs, with their search for evidence peppered with general, derogatory comments about French hygiene. In contrast, the mismatched pair’s deliberation about the same Tweet, included numerous loops and increasingly detailed investigation of the reliability of the sources on which the Tweet was based.

**Figure 11:**
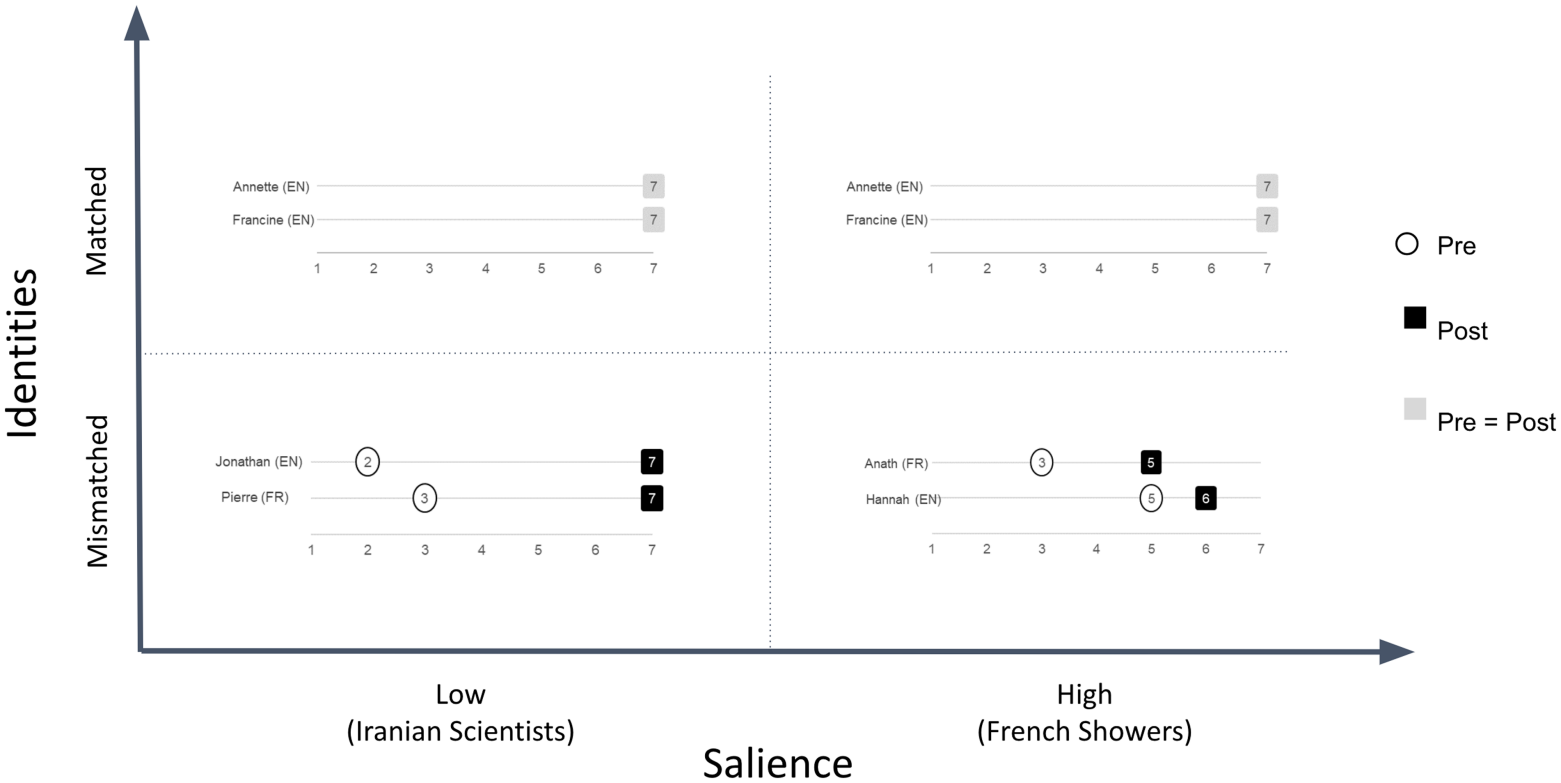
Belief change data for each of the deliberations analyzed in Figure 10.

These effects of content salience and interlocutor identity on fact-checking deliberations are further underlined by the belief change data for the four deliberations analyzed in Figure 11.

In the matched cases, salience had no effect on belief change. In contrast, both mismatched pairs exhibited belief change. However the change was much greater in the low salience case (Pierre and Jonathan on Iranian Scientists) than in the high salience case (Anath and Hannah on French Showers). Specifically, Jonathan and Pierre’s deliberation about the Iranian Scientists Tweet led them to revise significantly their assessments of its credibility: from 3 to 7 in Pierre’s case and 2 to 7 in Jonathan’s case. However, Anath and Hannah’s deliberation about the French Showers Tweet led them to revise their judgments more modestly – from 3 to 5 in Anath’s case and from 5 to 6 in Hannah’s case.

Taken together, these findings suggest that when the content of online information is of low identity-salience, the identities of the interlocutors have little effect on the deliberation, and belief change is a function primarily of evaluation of relevant evidence. However, when the content is of high-identity salience, the identities of the interlocutors significantly affect the deliberation. Specifically, matched pairs’ beliefs remain relatively impervious to the evidence, whereas mismatched pairs’ beliefs change in the direction of the evidence, but to a lesser degree than when the content is of low salience.

In summary, the data are suggestive of the hypothesized two-factor model that motivated our 2 × 2 design, namely that identity considerations set limits to fact-checking of online information, but neither replace nor eliminate it.

## 6. Discussion

### 6.1. Key findings

Our goals in this study were to investigate what leads people to fact-check online information; how they fact-check in practice; how such fact-checking affects their judgments about the information’s credibility; and how the above processes are affected by the salience of the online information to readers’ cultural identities and collaborative fact-checking with people whose identities differ from their own.

Within the limits of a case-study approach, our results suggest that people are led to fact-check online information when they perceive that information to be plausible but suspect, i.e., on the cusp of believability but neither obviously true nor obviously false. However, this alone is not sufficient to motivate fact-checking. To make the effort to search for, and evaluate, relevant evidence, people have first to conclude that the information is important enough — in general or personally — to warrant further investigation. If they consider the information suspect but trivial, they are less likely to fact-check it.

One factor that appears to be particularly relevant to people’s decisions about whether or not to fact-check online information is the salience of the information to their cultural identities. Whether to confirm stereotypes about relevant “Others,” as in Annette and Francine’s decision to fact-check a Tweet about how often French people shower, or to defend their own cultural group, as in Anath’s decision to fact-check the same Tweet, participants in our study often chose to fact-check Tweets that pertained in some way to their own cultural identities.

These findings should be considered suggestive rather than conclusive. It is likely that participants’ interest in issues of cultural difference were exaggerated in our study. After all, we recruited them explicitly to take part in a study of “how people from different backgrounds” assess the credibility of what they read online. Either out of social desirability (i.e., seeking to give the investigators what they want) or simply in response to an experimental situation in which they are invited to deliberate with someone from a different cultural background, they may have been motivated to focus more on issues of cultural identity and cultural difference than they would, had they been left to their own devices. Nevertheless, the fact that such reasons were given for checking some tweets and not others suggests that the presence of identity-salient content is indeed one motivator to fact-check.

With respect to the process by which people fact-check online information, our empirical findings suggest a novel account (summarized in Figure 5) wherein fact-checking deliberations comprise a determinate set of elements, which are deployed to differing extents, and in differing orders, depending on content and context. This empirically-generated model of online fact-checking differs from previous, theoretical models in at least two, important ways.

First, it posits (*contra* Metzger, 2007) that judgments of the credibility of online information do not occur only at the end of a fact-checking deliberation. Rather, readers make an initial assessment of the information’s credibility to decide whether or not it is worthy of further investigation. They then revise this initial assessment iteratively over the course of their deliberation before reaching a final judgment.

Second, these iterations can include loops, in which readers pass several times through the process of planning a search, formulating search terms, and interpreting search results, as they refine their conceptions of relevance and seek more precise forms of evidence. They can also include jumps, wherein readers skip from one element of fact-checking to another, apparently missing out intervening steps. This happened, for example, when some of our participants typed search terms into Google, pressed search, and interpreted the results, all in a matter of seconds, without saying anything about what they were doing while they were doing it.

Pre-post comparisons of participants’ assessments of Tweet credibility before and after deliberation indicate that the above fact-checking processes affect credibility judgments and lead to belief change. However, contrary to our expectations, such belief change was not exclusively, or even mostly, toward greater skepticism. For all but one of the Tweets included in the present study, there was movement in both directions following deliberation, with some participants considering Tweets more credible after deliberation and others considering them less credible after deliberation.

Moreover, these belief change effects were not limited to the specific Tweets that participant pairs discussed in practice. Rather, participants’ post-deliberation credibility judgments differed from their pre-deliberation judgments even regarding Tweets that they had not discussed. This suggests that fact-checking of online information has a general effect on the tendency to make certain types of credibility judgments, beyond the specific information checked.

Nevertheless, this latter effect may be somewhat exaggerated in the present study due to the fact that the Tweet fact-checked in practice by most participants was Iranian Scientists. This was a Tweet about which most participants (63%) were skeptical prior to deliberation and for which evidence supporting its credibility turned out, in practice, to be readily available. This may have led participants to adopt, following deliberation, a general expectation (or “mindset”) that their skepticism about other Tweets, too, might prove unfounded. If so, this might account for some of the positive belief change with respect to the other Tweets that they had not in fact checked. However, this potentially confounding factor is mitigated by the fact that, as noted earlier, many participants (ranging from 25 to 57%, depending on the Tweet) became more skeptical following deliberation, as opposed to less so.

Beyond the above insights into general processes of online fact-checking and their effect on belief change, our findings suggest that the salience of online information to the cultural identities of readers affects systematically both how they fact-check it and the degree to which such fact-checking leads them to revise their initial judgments of its credibility.

When salience is low (as in the case of the Iranian Scientists Tweet), fact-checking processes are simpler and more linear than when salience is high, and belief change is broadly in accord with the evidence discovered. This pattern appears to apply equally to matched and mismatched pairs.

However, when salience is high, we observe different patterns of fact-checking for matched and mismatched pairs. Specifically, when mismatched pairs fact-check online information of high identity-salience (such as when an English-French pair fact-checks a Tweet about how often French people shower), their deliberations tend to be longer and loopier than deliberations (whether by matched or mismatched pairs) about low salience content (such as Iranian Scientists). They also tend to be longer and loopier than the deliberations of matched pairs about high salience content (such as when an English-English pair fact-checks a Tweet about how often French people shower).

Moreover, these divergent patterns of fact-checking are associated with systematic differences in the extent and direction of belief change. Specifically, when matched pairs fact-check online information of high salience, they tend to confirm their initial beliefs rather than revise them. Mismatched pairs do the opposite: They revise their initial beliefs in the direction of the discovered evidence, even when the object of their fact-checking is of high salience. However, such revisions tend to be smaller than when the object of their fact-checking is of low salience.

Our results suggest that identity may affect fact-checking in several, related ways. First, it affects people’s motives to check or not to check a given piece of online information. Second, it affects the standards of credibility people apply to the evidence they discover pertaining to that information. Recall, for example, the suspicions of Anath, a French participant, about the motives of the opinion pollsters, and indeed of Google, when fact-checking the French Showers Tweet. This skepticism contrasted with the readiness of her English search partner, Hannah, to accept the evidence they encountered at face value.

Third, identity constrains belief change. When the online information investigated is of high identity-salience, matched pairs tend to confirm their initial beliefs after fact-checking. Mismatched pairs, on the other hand, tend to revise their belief in the direction of the evidence. But they do so to a lesser extent than occurs when the online information is of low salience.

### 6.2. Qualifications

While these findings are highly suggestive, they should be interpreted with caution. This was an exploratory study, in which we developed new methods of data collection and analysis to investigate empirically the role of cultural identity in processes of fact-checking.

Our decision to focus on collaborative fact-checking had the advantage of making people’s deliberations about the credibility of online information visible (audible). Because they were fact-checking together online, using a shared screen, our participants were required to make their thinking explicit, as they planned their search, formulated terms, and so on. This explicitness included not only the verbal interactions between them but also their use of the keyboard and their body language (albeit from the shoulder up) as they viewed their screens and considered the material before them.

However, a disadvantage of this focus on collaboration is that it introduces additional factors to the process of fact-checking that may be theoretically extraneous or perhaps even confounding. For example, collaborative interactions are rarely symmetrical. More commonly, one of a pair will do more heavy lifting than the other. Especially in mismatched pairs fact-checking highly salient online information, one participant may have greater knowledge of the context than the other. For example, a French participant in an English-French pair assessing the credibility of a statement attributed to Marine Le Pen might intuit immediately what makes the Tweet more or less credible, while their English search partner is still trying to figure out who Marine Le Pen is.

Language is another source of asymmetry. Depending on the Tweet and the participants, one member of a pair might be searching in their mother tongue while the other is searching, and communicating with their partner, in their second or third language. There are also individual differences between participants. Some people are more garrulous or ‘pushy’ than others, and tend to talk, or to take the lead in joint activities, more than others do.

Beyond these cultural, linguistic, and personal sources of asymmetry, our design included a further asymmetry in the division of labor between the designated searcher and their partner. By ‘owning’ the means of production (i.e., the keyboard), the power of the searcher to set the terms of the search and to follow up particular leads by clicking on them was greater, by default, than that of their search partners.

In the present study, we sought to address some of these asymmetries directly. For example, to minimize linguistic asymmetry, we produced English and French versions of the questionnaire, formed pairs that shared at least one common language and enabled them to interact in whichever of the two languages they felt most comfortable. Similarly, to minimize design asymmetry, we had pairs discuss two Tweets and switch roles: One participant being the searcher of the first deliberation, the other switching them out for the second deliberation. In practice, we did not observe any systematic differences between the processes or outcomes of a pair’s first deliberation versus their second deliberation. This suggests that, in the present study, which member of the pair “led” the deliberation was not a significant factor in determining the process or outcome of that deliberation.

However, it is not possible in a study of this kind to anticipate and eliminate all forms of asymmetry. Accordingly, it is important to bear in mind when interpreting our findings that collaborative fact-checking is a joint production and that how a participant fact-checked with this particular partner might differ from how they would fact-check with another partner or on their own. Most obviously and importantly, one cannot generalize safely from findings about collaborative fact-checking to conclusions about individual fact-checking.

Some additional qualifications are necessary regarding our operational definitions of cultural identity and identity-salience. Because pairs were free to choose to fact-check any two out of the six tweets presented in the questionnaire, we did not know in advance which dimensions of cultural identity or identity-salience (nationality or gender) would yield the most pertinent data to our investigation. In practice, we did not observe any significant gender effects.

In this context, it is important to remember that the notion of identity-salience is relative rather than absolute. For example, the question of how often French people shower can be assumed to be highly salient to French people. But because French people’s alleged poor hygiene is a common cultural stereotype in England, it is also salient to English people – for whom the French (like the Germans and the Irish) are a highly salient “Other.” This high identity-salience of the French Showers Tweet for English and French participants can be contrasted with the relatively low identity-salience for these participants of the Iranian Scientists Tweet. The gender distribution of science and engineering graduates in Iran is not an issue of special salience to English and French participants. We hypothesized that it might be more salient to women participants than to men participants. But we did not find any compelling evidence that this was the case in practice.

In future studies, we recommend that contrasts between high and low salience online information be sharpened further by selecting ‘hotter’ topics, such as ethnic conflicts, border disputes, and so on, about which people from different cultural backgrounds are more likely to diverge.

### 6.3. Implications

In this study, we developed a novel approach to investigating how people fact-check online information, and devised new methods for analyzing online fact-checking processes. This approach focuses on designing ecologically valid situations in which people engage in collaborative fact-checking. The advantage of this approach is that it makes deliberation visible, and therefore analyzable, as opposed to activity that is presumed to occur silently in individual heads.

A second novelty was our development of an empirical account of how people fact-check online information in practice. This account diverges in intriguing ways from previous, theoretical accounts. For example, rather than credibility judgments coming at the conclusion of a fact-checking process, we found them to occur iteratively over the course of a fact-checking deliberation.

A third novelty of our approach is the introduction of cultural identity – as both a motive and a constraint – in the investigation of fact-checking processes. Our comparisons of fact-checking processes and belief change under conditions of high and low identity-salience, and with pairs matched and mismatched for cultural identity, offer new methodological tools for investigating the role of cultural identity in fact-checking. Moreover, our findings suggest that cultural identity is an important factor in evaluation of online information, and thus warrants further, detailed study. Whereas the present study focused on national identity, future studies would be relevant on other dimensions of cultural identity, such as religion and ethnicity.

Beyond these methodological and theoretical advances, our findings suggest intriguing educational possibilities. If mismatched pairs are able to engage in extended deliberation about identity-salient online information, and to revise their beliefs in the direction of the evidence as a result, joint fact-checking by mismatched pairs may hold potential as a tool for teaching people to think critically about online information, even when that information impinges on their own cultural identities. In these times of misinformation, identity politics and polarization, such tools may be more crucial than ever.

## Data availability statement

The raw data supporting the conclusions of this article will be made available by the authors, without undue reservation.

## Ethics statement

Ethical approval was not required for the studies involving humans because participants were all legal adults, aged 30 and above, who provided written consent to participation in the study. The studies were conducted in accordance with the local legislation and institutional requirements. The participants provided their written informed consent to participate in this study.

## Author contributions

EG: Conceptualization, Data curation, Formal analysis, Investigation, Methodology, Validation, Visualization, Writing – original draft, Writing – review & editing. MB: Conceptualization, Data curation, Formal analysis, Funding acquisition, Investigation, Methodology, Project administration, Resources, Supervision, Validation, Visualization, Writing – original draft, Writing – review & editing. FD: Conceptualization, Formal analysis, Methodology, Validation, Visualization, Writing – original draft, Writing – review & editing.
